# A protocol for a systematic review of clinical practice guidelines for recurrent miscarriage

**DOI:** 10.12688/hrbopenres.13024.3

**Published:** 2020-10-02

**Authors:** Marita Hennessy, Rebecca Dennehy, Sarah Meaney, Declan Devane, Keelin O'Donoghue

**Affiliations:** 1 Pregnancy Loss Research Group, Department of Obstetrics and Gynaecology, University College Cork, Cork, Ireland; 2 The Irish Centre for Maternal and Child Health, University College Cork, Cork University Maternity Hospital, Cork, Ireland; 3 College of Medicine and Health, University College Cork, Cork, Ireland; 4 National Perinatal Epidemiology Centre, University College Cork, Cork University Maternity Hospital, Cork, Ireland; 5 School of Nursing and Midwifery, National University of Ireland, Galway, Galway, Ireland; 6 Evidence Synthesis Ireland, National University of Ireland, Galway, Galway, Ireland

**Keywords:** recurrent miscarriage, miscarriage, early pregnancy loss, systematic review, clinical guidelines, antenatal, care quality

## Abstract

Recurrent miscarriage (RM) was recently re-defined by the European Society of Human Reproduction and Embryology (ESHRE) as the loss of two or more consecutive pregnancies. Before this, and indeed still in some countries, RM was defined as three or more consecutive pregnancy losses. While the incidence of RM depends on the definition employed and population studied, it is generally accepted to affect 1-6% of women of reproductive age. Clinical practice guidelines (CPGs) for RM have been published by some professional organisations. While there are CPGs on miscarriage in Ireland, there are none concerning RM specifically. The aim of this systematic review is to identify, appraise and describe published CPGs for the management, investigation and/or follow-up of RM within high-income countries. Electronic databases (MEDLINE (Ovid ^®^; 1946), Embase ^®^ (Elsevier; 1980), CINAHL Complete (EBSCOhost; 1994), Web of Science™ (Thomson Reuters), Scopus (Elsevier; 2004), and Open Grey (INIST-CNRS; 2011)), selected guideline repositories, and the websites of professional societies will be searched to identify CPGs, published within the last 20 years, for potential inclusion. Two reviewers will review abstracts and full texts independently against the eligibility criteria. Characteristics and recommendations of included CPGs will be extracted by one reviewer and double-checked by another. Two reviewers will use the Appraisal of Guidelines for Research and Evaluation version 2 (AGREE II) instrument independently to assess the quality of the included CPGs. Narrative synthesis will be conducted to appraise and compare CPGs and their recommendations or guidance therein. The identification, appraisal and description of published CPGs in other high-income countries will be a valuable first step in informing efforts to promote the optimisation and standardisation of RM care.

## Abbreviations

AGREE, Appraisal of Guidelines for Research and Evaluation; CPG, Clinical Practice Guideline; ESHRE, European Society of Human Reproduction and Embryology; NCEC, National Clinical Effectiveness Committee; PRISMA, Preferred Reporting Items for Systematic Reviews and Meta-Analyses; PROSPERO, International Prospective Register of Systematic Reviews; RM, Recurrent Miscarriage; SIGN, Scottish Intercollegiate Guidelines Network; WHO, World Health Organisation

## Introduction

Recurrent miscarriage (RM) was recently re-defined by the European Society of Human Reproduction and Embryology (ESHRE) as the loss of two or more consecutive pregnancies ^1^. Before this, it was defined as three or more consecutive pregnancy losses, and this definition is still in use in some countries, including the UK ^2^. The actual term used to describe the condition can also vary between countries and/or professional bodies; for example, ESHRE uses the term ‘recurrent pregnancy loss’ ^1^, while the Royal College of Obstetricians and Gynaecologists uses the term RM ^2^. While the incidence of RM depends on the definition employed and population studied, it is generally accepted to affect 1–6% of the reproductive age population ^1, 3^. Given that 6% of women experience two or more consecutive miscarriages, more women will be accessing services for investigation and management as the new definition of RM is adopted internationally ^1, 4^.

There is a need for consistent clinical care of RM that follows the best evidence-based practice. Previous reproductive history is an independent predictor of future pregnancy outcome. The risk of a further miscarriage increases after each successive pregnancy loss, reaching approximately 40% after three consecutive pregnancy losses, and the prognosis worsens with increasing maternal age ^5, 6^. A previous live birth does not prevent a woman experiencing RM ^5, 6^. There are a few common established biological causes of miscarriage ^2, 7^, along with some more recent proposed aetiologies ^7, 8^, which are still controversial. However, a high proportion, even when recurrent, are classified as unexplained. Despite this, the standard investigations for RM continue to be important in evaluating potential factors responsible for pregnancy loss ^9^, and similar procedures are included in all international clinical practice guidelines (CPGs) ^1, 7^. While some evidence-based treatments have improved the outcomes for couples with RM, almost half of cases remain unexplained and are empirically treated ^9^. While future pregnancy may be difficult, the likelihood of subsequent live birth is approximately 70–75% ^5, 10^. The psychological impact of RM can be both severe and protracted, and studies indicate that it can negatively affect both men’s and women’s psychological well-being in the medium- to long-term ^11^. Studies have indicated that 32% of women with RM could be classified as depressed, against which having a living child was not a protective buffer ^12^. Thus, given its high frequency, RM can significantly contribute to the overall burden of psychopathology within a population, and recognition of this impact is important, so that affected individuals may be cared for appropriately ^13– 15^.

CPGs are “statements that include recommendations intended to optimise patient care that are informed by a systematic review of evidence and an assessment of the benefits and harms of alternative care options” ^16^. CPGs for the management, investigation, and/or follow-up of those who experience RM have been issued by professional societies such as ESHRE ^1^, the American College of Obstetricians and Gynaecologists ^17^, and the Royal College of Obstetricians and Gynaecologists ^18^; however, the existence of guidelines in other high-income countries is unknown to the study team. While there are CPGs on miscarriage in Ireland ^19, 20^, there are none concerning RM specifically. The identification, appraisal and description of published CPGs in other high-income countries would be a valuable first step in informing efforts to promote the optimise and standardise RM care.

### Aim of this review

The aim of this systematic review is to identify, appraise and describe published CPGs for the management, investigation and/or follow-up of RM within high-income countries.

***Objectives***

- To identify published CPGs for the management, investigation and/or follow-up of RM within high-income countries;
- To appraise the quality of included CPGs using the Appraisal of Guidelines for Research and Evaluation version 2 (AGREE II) instrument;
- To describe recommendations from the included CPGs concerning first trimester RM.

## Protocol

Details of the review have been submitted for registration to the PROSPERO database (ID: 173881). Any protocol amendments will be noted on PROSPERO and in any publications arising from the study. This protocol follows the PRISMA-P guidelines for the reporting of systematic review protocols ^21^; the completed checklist is available as Extended Data ^22^. Methodological guidance on conducting systematic reviews of CPGs was also followed in the preparation of this protocol, as such reviews require tailored approaches to, and greater subjectivity in, design and execution compared with other systematic reviews ^23^.

### Eligibility criteria

The ‘‘PICAR’’ framework was used to guide review inclusion and exclusion criteria ( Table 1). For the purpose of this review, CPGs are defined as “systematically developed statements to assist practitioners about appropriate health care for specific clinical circumstances”; an adaptation of the definitions used by National Clinical Effectiveness Committee (NCEC) ^24^ and Scottish Intercollegiate Guidelines Network (SIGN) ^32^.

**Table 1.** PICAR statement.

PICAR framework	Eligibility criteria
**P**opulation, clinical indication(s), and condition(s)	**Study population** • Women/couples experiencing recurrent miscarriage (RM) • Humans only **Clinical indication** • Investigation, management and/or follow-up of women/couples with RM – specifically first trimester RM **Clinical condition** • RM; defined by the review team as the loss of two or more consecutive pregnancies ^1^, with a specific focus on first trimester RM. For the purposes of this review, all CPGs that focus on RM – regardless of the definition used – will be included. The definition applied by each included CPG will be extracted and considered when synthesising and interpreting the review findings
**I**nterventions	• Any intervention focusing on the investigation, management and/or follow-up of RM
**C**omparator(s), Comparison(s), and (key) Content	• Any comparator or comparison • No ‘key’ CPG content is of interest – unless CPGs are broader in scope; in such instances, content specific to RM is only of interest
**A**ttributes of eligible CPGs	**Language** • Available in English • CPGs where summaries are available in English, but full text is not, will be excluded **Year of publication** • 2000 onwards • In Ireland, the National Clinical Effectiveness Committee (NCEC), requires a full guideline update within three years ^24^; while The Scottish Intercollegiate Guidelines Network (SIGN) also specifies three years, it also includes those over three years old and revalidated ^25^. The World Health Organisation does not have a defined period for guideline updates ^26^. To be comprehensive, CPGs published within the last twenty years (January 2000 to date) will be eligible for inclusion given that international CPGs concerning RM can fall well outside the three-year period ^17, 27^. A good quality older guideline could be a good base on which to develop a new guideline ^28^ **Developing/publishing organisation** • Only CPGs issued or endorsed by national or international scientific societies, professional colleges, charitable organisations, and government organisations will be included **Country of publication** • High-income countries, as defined by the World Bank ^29^; given the large discrepancies in pregnancy outcomes and care structures between high, and low and middle-income countries ^30, 31^ **Version** • Latest version only **Development process** • Evidence and/or consensus-based **System of rating evidence** • Use of a system to rate the level of evidence within CPGs is not an eligibility criterion; however, such data will be extracted to inform synthesis and interpretation of findings **Quality of evidence** • The eligibility of CPGs will not be based on a specific minimum quality cut-off score based on the AGREE II criteria. • We are interested in all guidance generated regardless of quality (e.g. because CPGs determined to be of ‘‘high quality’’ may not necessarily report recommendations that are highly valid and implementable ^23^); this will however be taken into consideration when synthesising and interpreting the review findings **Scope** • Must have a primary/secondary focus on the investigation and treatment of RM • Must be national/international in scope • Covers any aspect of RM care and its organisation; including the provision of dedicated pregnancy loss clinics, treatment and management of RM, investigations performed following RM in order to inform prognosis of future pregnancy outcomes, and counselling of parents following RM • Must be clearly identified as a CPG • Must be published. Unpublished CPGs, conference papers, discussion papers, drafts and opinions will be excluded **Recommendations** • Must have ‘recommendations’ concerning the identification, management and/or follow-up RM (either explicitly highlighted as such within the document, or noted within the body of the document, but not explicitly identified as a recommendation) • To be eligible, recommendations need not be accompanied by an explicit level of confidence (and quality assessment criteria system used specified); however, this data will be extracted (where available) and considered during the synthesis and interpretation of findings

### Information sources

While many CPGs are published in journals and can be identified through systematic bibliographic database searching, others may only be published in non-commercial or proprietary formats and are accessible only through extensive searches of grey literature sources or posted by professional medical associations on their websites behind membership paywalls ^33^. We will therefore use a range of information sources to locate CPGs concerning RM.

A systematic literature search, covering CPGs published from 2000 to present, will be performed using the following databases: MEDLINE (Ovid ^®^; 1946), Embase ^®^ (Elsevier; 1980), CINAHL Complete (EBSCOhost; 1994), Web of Science™ (Thomson Reuters), Scopus (Elsevier; 2004), and Open Grey (INIST-CNRS; 2011). Guideline repositories ( Table 2) and the websites of professional organisations/associations from around the world ( Table 3) will also be searched. Searches of Web of Science, Scopus and Open Grey, as well as guideline repositories and the websites of professional bodies/organisations, will facilitate the identification of grey literature – such as conference proceedings and/or technical reports – which may contain information about potentially eligible CPGs.

**Table 2.** Information sources: Guideline repositories.

Guideline repositories	Website
Canadian Agency for Drugs and Technology in Health (CADTH)	www.cadth.ca
Guidelines International Network (GIN)	http://www.g-i-n.net/library/international-guidelines-library
Institute for Clinical Systems Improvement (ICSI)	www.icsi.org/guidelines
Lenus: The Irish Health Repository	https://www.lenus.ie/hse/
National Institute for Health and Care Excellence (NICE)	www.nice.org.uk
Scottish Intercollegiate Guidelines Network (SIGN)	http://www.sign.ac.uk/index.html
TRIP database	https://www.tripdatabase.com
World Health Organisation	https://www.who.int/publications/guidelines/en/

**Table 3.** Information sources: Professional bodies/organisations.

Organisation	Country/Region
Royal College of Physicians of Ireland Institute of Obstetricians and Gynaecologists	Ireland
Royal College of Obstetricians and Gynaecologists (RCOG)	UK
European Society of Human Reproduction and Embryology (ESHRE)	Europe
The International Federation of Gynaecology and Obstetrics (FIGO)	International
The American College of Obstetricians and Gynaecologists (ACOG)	US
The Royal Australian and New Zealand College of Obstetricians and Gynaecologists (RANZCOG)	Australia & New Zealand
American Society for Reproductive Medicine (ASRM)	US
Society for Maternal-Fetal Medicine (SMFM)	International
Society of Obstetricians and Gynaecologists of Canada (SOGC)	Canada

### Search strategy

This search strategy was developed with the assistance of a specialist librarian. Key word searches, using combinations of key words and Medical Subject Headings (or equivalent), will be used across two concepts using the AND Boolean operator: (1) clinical guidelines; (2) recurrent miscarriage ( Table 4). Within each of the categories, keywords will be combined using the “OR” Boolean operators. The search strategy will be developed in Medline (see Extended Data for sample search strategy ^22^ and tailored for use within the other databases, and piloted, before final searches are run.

**Table 4.** Search terms.

Concept	Search terms
1: Clinical guidelines	guideline* OR standard* OR best practice* OR guidance
2: Recurrent miscarriage	Miscarriage* OR pregnancy loss* OR spontaneous abortion* OR recurrent fetal loss* OR recurrent foetal loss*

### Study records

***Data management.*** Records will be imported into EndNote X9 and de-duplicated using the ‘remove duplicates’ function, as well as manually screening results for accuracy.

***Selection process.*** Two independent reviewers (MH and RD) will screen titles and abstracts of retrieved records against the inclusion criteria. Records not meeting the eligibility criteria will be excluded. Two reviewers (MH and RD) will subsequently, and independently, screen the full text articles of records identified to identify studies to be included. Any disagreements in eligibility assessments will be discussed and resolved via consensus. If consensus on eligibility cannot be agreed between the two reviewers, a third reviewer (KOD) will review the particular record(s) in order to determine its eligibility of the CPG.

A Preferred Reporting Items for Systematic Review and Meta-Analysis (PRISMA) flow diagram will show the overall process of CPG selection and summarise the inclusion and exclusion of records/CPGs at each stage of the review.

***Data collection process.*** Once the final set of included CPGs has been obtained, all documents related to the CPGs (cited as supplemental documents, summaries of recommendations, and others) will be retrieved by MH before data extraction or quality assessment is undertaken. If links to these documents are not provided in the included CPG, MH will conduct searches to locate them. For CPGs published only in summary or where important information is missing, we will try to find complete information by contacting the authors. All documents collected will be verified independently by RD to confirm completeness and to ensure that companion documents are matched appropriately. MH will also conduct searches to ensure that the latest version of each included CPG has been included, and none is present in duplicate.

### Data items

Key features of CPGs and recommendations, for all included CPGs, will be extracted using a structured data extraction form in Microsoft Excel (Microsoft Corporation, Redmond, WA) (see template in Extended Data ^22^), which will be piloted in advance.

Key features of CPGs to be extracted, include:

- Title
- Year of publication
- Language
- Developing/publishing organisation and/or authors
- Country/countries of publication
- How described by the authors (e.g. guideline / standard)
- Version
- Type of CPG (formulated, adapted, updated or revised)
- Topic addressed (i.e. RM or broader)
- Development process (evidence- and/or expert consensus-based)
- Composition of guideline development group
- Peer-review conducted, or not
- Target users
- Definition of RM employed – to include number of miscarriages, whether consecutive/not, number of weeks gestation
- System of rating evidence/Quality instrument used during CPG development (GRADE, Oxford, not mentioned, or other), if any – some developers do not include levels of evidence with their recommendations ^28^
- All recommendations related to first trimester RM within the CPG.

Data will be extracted by one reviewer (MH) and independently verified for accuracy and completeness by a second reviewer (SM), with discrepancies resolved through consensus. If agreement cannot be reached, a third reviewer (KOD) will review and make a final decision. If a member of the review team has been involved in the development of any of the CPGs eligible for the review, an independent reviewer will extract the required data from the study.

### Outcomes and prioritisation

Not applicable.

### Risk of bias in individual studies/quality assessment

The quality of included CPGs will be assessed using the Appraisal of Guidelines, Research and Evaluation version 2 (AGREE II) criteria ^34^. The criteria encompass 23 items, over six domains, rated on a 7-point Likert scale: (i) Scope and purpose of the guideline; (ii) Stakeholder involvement in the development of the guidelines; (iii) Rigour of development and formulation of the recommendations within the guideline; (iv) Clarity of presentation of the guideline; (v) Applicability of the guideline; (vi) editorial independence in the formulation of recommendations within the guideline. As part of the overall assessment, two global ratings are included: (i) a rating on the overall quality of the guideline, and (ii) whether the guideline would be recommended for use in practice. AGREE II is an accepted and validated tool for assessing the methodological quality of CPGs ^35^. It has limitations, however; for example, it does not assess the implementation of the guideline ^36^.

Two reviewers with methodological and clinical expertise (MH/SM and KOD) will conduct an independent quality assessment of the CPGs. Domain scores will be calculated by summing up all the scores of the individual items in a domain and by scaling the total as a percentage of the maximum possible score for that domain, as per the AGREE II User Manual. The six domains are independent, and the scores will be calculated as the sum of the individual items in each domain.

To make the scores more relevant to readers and enable fair comparison, our review will report the AGREE II outcomes categorically rather than statistically, using the 5-point Likert scale described by other reviews ^36, 37^: excellent (>80%), good (>60%–80%), average (>40%–60%), fair (>20%–40%) and poor (≤20%).

### Data synthesis

A narrative synthesis approach will be used to describe and appraise CPGs and their recommendations or guidance therein, taking account of quality appraisal (using the AGREE II tool), and recency of publication. The levels of evidence associated with the recommendations within each CPG will be reported, and quality assessment rating system used; we will not attempt to standardise evidence ratings across CPGs.

### Dissemination

The Preferred Reporting Items for Systematic Reviews and Meta-Analyses (PRISMA) checklist ^38^ will be used to report findings of the review, as there is currently no specific checklist for systematic reviews of CPGs. We will share the findings in a peer-reviewed journal, through communications with professional bodies and policymakers (through briefings), and participation in scientific meetings and national and international conferences.

### Patient and public involvement (PPI)

This systematic review protocol was developed in conjunction with a Pregnancy Loss Patient Representative and through consultations with Specialist Bereavement and Loss Midwives. A PPI group is currently being established and will have input into discussions and decisions concerning the conduct, findings and outputs of this review.

## Study status

Database searches have been completed.

## Conclusions/discussion

CPGs for RM have been published by some professional organisations. In Ireland, there is currently no national standard for the management, investigation or follow-up of those who experience RM. The aim of this systematic review is to identify, appraise and describe published CPGs for the management, investigation and/or follow-up of RM within high-income countries. This will be a valuable first step in informing efforts to promote the optimisation and standardisation of the management, investigation and follow-up of RM.

## Data availability

### Underlying data

No data are associated with this article.

### Extended data

Open Science Framework: A systematic review of clinical practice guidelines for recurrent miscarriage, https://doi.org/10.17605/OSF.IO/X7Y4N ^22^.

This project contains the following extended data:

- Supplementary File 1. PRISMA-P checklist for the reporting of systematic review protocols
- Supplementary File 2. Sample search strategy
- Supplementary File 3. Data extraction form template

Data are available under the terms of the Creative Commons Zero "No rights reserved" data waiver (CC0 1.0 Public domain dedication).

## References

[1] ESHRE Early Pregnancy Guideline Development Group: Guideline on the Management of Recurrent Pregnancy Loss. Version 2.Grimbergen, Belgium: European Society of Human Reproduction and Embryology.2017 Reference Source

[2] Royal College of Obstetricians and Gynaecologists: Green-top Guideline Number 17. The Investigation and Treatment of Couples with Recurrent First-trimester and Second-trimester Miscarriage. London: Royal College of Obstetricians and Gynaecologists;2011 Reference Source

[3] Roman E: Fetal loss rates and their relation to pregnancy order. *J Epidemiol Community Health.* 1984;38(1):29–35. 10.1136/jech.38.1.29 6707560 PMC1052312

[4] Rai R Regan L: Recurrent miscarriage. *Lancet.* 2006;368(9535):601–11. 10.1016/S0140-6736(06)69204-0 16905025

[5] Clifford K Rai R Regan L: Future pregnancy outcome in unexplained recurrent first trimester miscarriage. *Hum Reprod.* 1997;12(2):387–9. 10.1093/humrep/12.2.387 9070732

[6] Nybo Andersen AM Wohlfahrt J Christens P: Maternal age and fetal loss: population based register linkage study. *BMJ.* 2000;320(7251):1708–12. 10.1136/bmj.320.7251.1708 10864550 PMC27416

[7] El Hachem H Crepaux V May-Panloup P: Recurrent pregnancy loss: current perspectives. *Int J Womens Health.* 2017;9:331–45. 10.2147/IJWH.S100817 28553146 PMC5440030

[8] Matjila MJ Hoffman A van der Spuy ZM: Medical conditions associated with recurrent miscarriage-Is BMI the tip of the iceberg? *Eur J Obstet Gynecol Reprod Biol.* 2017;214:91–6. 10.1016/j.ejogrb.2017.05.003 28494269

[9] Clifford K Rai R Watson H: An informative protocol for the investigation of recurrent miscarriage: preliminary experience of 500 consecutive cases. *Hum Reprod.* 1994;9(7):1328–32. 10.1093/oxfordjournals.humrep.a138703 7962442

[10] Brigham SA Conlon C Farquharson RG: A longitudinal study of pregnancy outcome following idiopathic recurrent miscarriage. *Hum Reprod.* 1999;14(11):2868–71. 10.1093/humrep/14.11.2868 10548638

[11] Lok IH Neugebauer R: Psychological morbidity following miscarriage. *Best Pract Res Clin Obstet Gynaecol.* 2007;21(2):229–47. 10.1016/j.bpobgyn.2006.11.007 17317322

[12] Klock SC Chang G Hiley A: Psychological distress among women with recurrent spontaneous abortion. *Psychosomatics.* 1997;38(5):503–7. 10.1016/S0033-3182(97)71428-2 9314720

[13] Farren J Mitchell-Jones N Verbakel JY: The psychological impact of early pregnancy loss. *Hum Reprod Update.* 2018;24(6):731–49. 10.1093/humupd/dmy025 30204882

[14] Tavoli Z Mohammadi M Tavoli A: Quality of life and psychological distress in women with recurrent miscarriage: a comparative study. *Health Qual Life Outcomes.* 2018;16(1):150. 10.1186/s12955-018-0982-z 30055644 PMC6064101

[15] Kolte AM Olsen LR Mikkelsen EM: Depression and emotional stress is highly prevalent among women with recurrent pregnancy loss. *Hum Reprod.* 2015;30(4):777–82. 10.1093/humrep/dev014 25662810 PMC4359400

[16] Institute of Medicine: Clinical Practice Guidelines We Can Trust.In: Graham RMM, Wolman DM, Greenfield S, Steinberg E, editor. Washington, DC: The National Academies Press;2011. 10.17226/13058 24983061

[17] American College of Obstetricians and Gynecologists: ACOG practice bulletin. Management of recurrent pregnancy loss. Number 24, February 2001. (Replaces Technical Bulletin Number 212, September 1995). American College of Obstetricians and Gynecologists. *Int J Gynaecol Obstet.* 2002;78(2):179–90. 10.1016/s0020-7292(02)00197-2 12360906

[18] Royal College of Obstetricians and Gynaecologists: Recurrent Miscarriage, Investigation and Treatment of Couples (Green-top Guideline No. 17).London: Royal College of Obstetricians and Gynaecologists;2011 Reference Source

[19] Institute of Obstetricians and Gynaecologists Royal College of Physicians of Ireland, Directorate of Strategy and Clinical Programmes Health Service Executive: Clinical Practice Guideline: Management of Early Pregnancy Miscarriage, Version 1.0.Dublin: Institute of Obstetricians and Gynaecologists Royal College of Physicians of Ireland;2012 Reference Source

[20] Institute of Obstetricians and Gynaecologists Royal College of Physicians of Ireland, Directorate of Strategy and Clinical Programmes Health Service Executive: Clinical Practice Guideline: The Management of Second Trimester Miscarriage, Version 1.0.Dublin: Institute of Obstetricians and Gynaecologists Royal College of Physicians of Ireland;2014 Reference Source

[21] Moher D Shamseer L Clarke M: Preferred Reporting Items for Systematic Review and Meta-Analysis Protocols (PRISMA-P) 2015 Statement. *Syst Rev.* 2015;4(1):1. 10.1186/2046-4053-4-1 25554246 PMC4320440

[22] Hennessy M Dennehy R Meaney S: A systematic review of clinical practice guidelines for recurrent miscarriage.2020 10.17605/OSF.IO/X7Y4N PMC7477641 33005862

[23] Johnston A Kelly SE Hsieh SC: Systematic Reviews of Clinical Practice Guidelines: A Methodological Guide. *J Clin Epidemiol.* 2019;108:64–76. 10.1016/j.jclinepi.2018.11.030 30529647

[24] National Clinical Effectiveness Committee: How to develop a National Clinical Guideline: A manual for guideline developers.Dublin: Department of Health;2019 Reference Source

[25] Scottish Intercollegiate Guidelines Network (SIGN): A guideline developer’s handbook.Edinburgh: Scottish Intercollegiate Guidelines Network;2019 Reference Source

[26] World Health Organisation: WHO handbook for guideline development –2nd ed.Geneva: World Health Organistaion;2014 Reference Source

[27] Association of Early Pregnancy Units: Guidelines. London: Association of Early Pregnancy Units;2007 Reference Source

[28] The ADAPTE Collaboration: The ADAPTE Process: Resource Toolkit for Guideline Adaptation. Version 2.0.Berlin: Guideline International Network;2009 Reference Source

[29] World Bank: World Bank Country and Lending Groups.2020 Reference Source

[30] Goldenberg RL McClure EM Saleem S: Improving pregnancy outcomes in low- and middle-income countries. *Reprod Health.* 2018;15(Suppl 1):88. 10.1186/s12978-018-0524-5 29945628 PMC6019988

[31] Gage AD Carnes F Blossom J: In Low- And Middle-Income Countries, Is Delivery In High-Quality Obstetric Facilities Geographically Feasible? *Health Aff (Millwood).* 2019;38(9):1576–84. 10.1377/hlthaff.2018.05397 31479351

[32] SIGN: What are guidelines?Edinburgh: SIGN;2020 Reference Source

[33] Methley AM Campbell S Chew-Graham C: PICO, PICOS and SPIDER: a comparison study of specificity and sensitivity in three search tools for qualitative systematic reviews. *BMC Health Serv Res.* 2014;14(1):579. 10.1186/s12913-014-0579-0 25413154 PMC4310146

[34] Brouwers MC Kho ME Browman GP: AGREE II: advancing guideline development, reporting and evaluation in health care. *CMAJ.* 2010;182(18):E839–42. 10.1503/cmaj.090449 20603348 PMC3001530

[35] Siering U Eikermann M Hausner E: Appraisal tools for clinical practice guidelines: a systematic review. *PLoS One.* 2013;8(12):e82915. 10.1371/journal.pone.0082915 24349397 PMC3857289

[36] Daley B Hitman G Fenton N: Assessment of the methodological quality of local clinical practice guidelines on the identification and management of gestational diabetes. *BMJ Open.* 2019;9(6):e027285. 10.1136/bmjopen-2018-027285 31201189 PMC6576117

[37] Eady EA Layton AM Sprakel J: AGREE II assessments of recent acne treatment guidelines: how well do they reveal trustworthiness as defined by the U.S. Institute of Medicine criteria? *Br J Dermatol.* 2017;177(6):1716–25. 10.1111/bjd.15777 28667760

[38] Moher D Liberati A Tetzlaff J: Preferred Reporting Items for Systematic Reviews and Meta-Analyses: The PRISMA Statement. *PLoS Med.* 2009;6(7):e1000097. 10.1371/journal.pmed.1000097 19621072 PMC2707599

