# Supplementary material for: A protocol for a systematic review of clinical practice guidelines for recurrent miscarriage
Source: HRB Open Res. 2020 Oct 2;3:12. Originally published 2020 Apr 1. [Version 3] doi: 10.12688/hrbopenres.13024.3 (PMC7477641; doi:10.12688/hrbopenres.13024.3)
Supplement: Peer review history

# Peer review: Reviewer response for version 3

**DOI:** 10.21956/hrbopenres.14282.r28055

**Published:** 2020-12-14

**Authors:** Emma Rasmark Roepke

**Affiliations:** 1 Department of Obstetrics and Gynecology, Institute of Clinical Sciences Lund, Skåne University Hospital, Lund University, Lund, Sweden

I find that the revisions are appropriate and sufficient to be Approved for indexing.

Is the study design appropriate for the research question?

Partly

Is the rationale for, and objectives of, the study clearly described?

Yes

Are sufficient details of the methods provided to allow replication by others?

Yes

Are the datasets clearly presented in a useable and accessible format?

Yes

Reviewer Expertise:

Recurrent pregnancy loss

I confirm that I have read this submission and believe that I have an appropriate level of expertise to confirm that it is of an acceptable scientific standard.

# Peer review: Reviewer response for version 1

**DOI:** 10.21956/hrbopenres.14119.r27802

**Published:** 2020-09-21

**Authors:** Matthew Coleman, Sarah Bailey

**Affiliations:** 1 University Hospital Southampton, University of Southampton, Southampton, UK

*This peer review report was updated on 22nd September 2020 to incorporate further comments from the reviewers.*

**Matthew Coleman:**

**Summary**

 A few comments to help guide the authors. The vision is laudable however I am uncertain about the process especially in this highly emotive area.

 Whilst the overall methods quote and follow national and international recommended methodology, the original challenge is made extremely complex by any number of human factors and biases. Current evidence is largely opinion based and confounded by bias and human factors. And given the distress caused by the disease these have sometimes been exaggerated. For example, the reasons behind the recent change in Rm definition, which might include as many as 6% of women, is complex and multi factorial and makes evaluating possible interventions difficult for the majority of women for whom the outlook is already consistent with women who have not experienced to consecutive miscarriages.

 Understanding the psychology and reasons behind the recommendations would provide much more useful background for developing future guidelines which are rational fair and achievable.

**Scope **There appears to be some conflict in the defined scope of this review;

**Scope**:

- **Must have a primary/secondary focus on the investigation/treatment** of RM.
- Must be national/international in scope.
- **Covers any aspect of RM care and its organisation.**
- Must be clearly identified as a CPG.
- Must be published.

**Time span;** given the rapid evolution of recommended managements in this particular area including recent and distant past guideline recommendations (as far back as 2000) any review is likely to produce significant inconsistencies related to change over time. Amalgamating them together will require some complexity to control for these changes, which are also destabilised by the paucity of data, bias and frequent personal opinion.

**Sarah Bailey:**

 The objective of this protocol for a systematic review of clinical practice guidelines (CPG) is to identify, appraise and describe published CPGs of the management, investigation and follow-up of recurrent miscarriage (RM) within high-income countries. The aim being to take the timely and important first step to promote consistent and evidence based care for couples with RM in Ireland.

 The protocol is well-written and easy to follow and the proposed methodology is, overall, clearly described using recognised frameworks and tools to assist.   

 Please see below comments;

 Introduction:

- In addition to highlighting that the definition of RM, with regards to number of miscarriages that constitute the term, varies from country to country, it would be useful to note at this point that actual term used to describe the condition can significantly vary between countries and CPGs – e.g. ESHRE uses term recurrent pregnancy loss (ESHRE 2017).

Protocol:

- The PICAR statement provides a useful and thorough framework to guide the review of inclusion and exclusion criteria, as clearly demonstrated in Table 1.
- Clarification is required of how the researchers plan to identify relevant ‘grey’ unpublished literature and I would advise the authors to make clear the exact range of information sources that will be utilised to locate CPGs concerning RM to allow a reproducible literature search.  However, the PICAR statement in Table 1, also identifies that unpublished CPGs will be excluded. This is confusing given the plan to locate unpublished literature sources and this inconsistency should be addressed. 
- I was pleased to see the protocol was developed in conjunction with a Pregnancy Loss Patient Representative and encourage the prompt establishment of a PPI group to support this research.

Conclusion:   

 I assume the overall aim of the systematic review is to develop a CPG on RM to promote consistent and standardized care for couples with RM in Ireland, but the actual planned outcome is not clearly articulated and it would be useful to have clarification of this.

Is the study design appropriate for the research question?

Partly

Is the rationale for, and objectives of, the study clearly described?

Yes

Are sufficient details of the methods provided to allow replication by others?

Yes

Are the datasets clearly presented in a useable and accessible format?

Not applicable

Reviewer Expertise:

Obstetric medicine, haematology and pregnancy, diabetes pregnancy, hypertension pregnancy. Clinical Specialist in Recurrent Miscarriage.

We confirm that we have read this submission and believe that we have an appropriate level of expertise to confirm that it is of an acceptable scientific standard, however we have significant reservations, as outlined above.

# Response

**Published:** 2020-10-02

**Authors:** Marita Hennessy

**Affiliations:** University College Cork, Ireland

***Matthew Coleman***

Comment 1:

A few comments to help guide the authors. The vision is laudable however I am uncertain about the process especially in this highly emotive area. Whilst the overall methods quote and follow national and international recommended methodology, the original challenge is made extremely complex by any number of human factors and biases. Current evidence is largely opinion based and confounded by bias and human factors. And given the distress caused by the disease these have sometimes been exaggerated. For example, the reasons behind the recent change in Rm definition, which might include as many as 6% of women, is complex and multi factorial and makes evaluating possible interventions difficult for the majority of women for whom the outlook is already consistent with women who have not experienced to consecutive miscarriages. Understanding the psychology and reasons behind the recommendations would provide much more useful background for developing future guidelines which are rational fair and achievable.

*Response:*

Thank you for taking the time to review and provide considered feedback on our protocol paper. We acknowledge the challenges in this area. As noted in the protocol, this study is a first step in informing efforts to promote the optimisation and standardisation of RM care. This review is being conducted as part of a larger project titled “REcurrent miscarriage: Evaluating CURRENT services (RE:CURRENT)” which is looking at service provision in Ireland. The findings from the systematic review will be used by the RE:CURRENT Team to inform this evaluation; however, we did not detail this within the protocol as the findings of this systematic review will have relevance to the field in general, not just the RE:CURRENT Team. A national guideline is not a planned output of this project ; however, evidence gathered during the project may aid future development of a national guideline.

Comment 2:

There appears to be some conflict in the defined scope of this review;

Scope:

- *Must have a primary/secondary focus on the investigation/treatment of RM.*
- Must be national/international in scope.
- *Covers any aspect of RM care and its organisation.*
- Must be clearly identified as a CPG.
- Must be published.

Time span; given the rapid evolution of recommended managements in this particular area including recent and distant past guideline recommendations (as far back as 2000) any review is likely to produce significant inconsistencies related to change over time. Amalgamating them together will require some complexity to control for these changes, which are also destabilised by the paucity of data, bias and frequent personal opinion.

*Response:*

Thank you for raising these important points. CPGs which focus specifically on RM, or address any aspect of RM care within a broader/different topic area (e.g. CPGs on pregnancy loss, or thrombophilia, which may have a section on RM), are eligible for inclusion.

While we are including CPGs published from 2000 onwards, we also state that CPGs must be ‘latest version only’ and note in the data collection process section that MH will conduct searches to ensure that the latest (i.e. current and valid) version of each included CPG has been included. We also state that when describing and appraising CPGs and recommendations therein in our narrative synthesis, we will take account of quality appraisal scores (as per the six domains within the AGREEII tool, including rigour of development and formulation of recommendations) and recency of publication. We will also extract systems of rating evidence used by CPGs and present these along with the recommendations.

***Sarah Bailey***

General comment

The objective of this protocol for a systematic review of clinical practice guidelines (CPG) is to identify, appraise and describe published CPGs of the management, investigation and follow-up of recurrent miscarriage (RM) within high-income countries. The aim being to take the timely and important first step to promote consistent and evidence based care for couples with RM in Ireland.  

The protocol is well-written and easy to follow and the proposed methodology is, overall, clearly described using recognised frameworks and tools to assist.  Please see below comments.

*General response*

Thank you for taking the time to review and provide considered feedback on our protocol paper.

Comment 1:

Introduction: In addition to highlighting that the definition of RM, with regards to number of miscarriages that constitute the term, varies from country to country, it would be useful to note at this point that actual term used to describe the condition can significantly vary between countries and CPGs – e.g. ESHRE uses term recurrent pregnancy loss (ESHRE 2017).

*Response:*

Thank you; we have added the following to the introduction: “The actual term used to describe the condition can also vary between countries and/or professional bodies; for example, ESHRE uses term ‘recurrent pregnancy loss’ ^1^, while the Royal College of Obstetricians and Gynaecologists uses the term RM ^2^. ”

Comment 2:

The PICAR statement provides a useful and thorough framework to guide the review of inclusion and exclusion criteria, as clearly demonstrated in Table 1.

*Response:*

Thank you; it was certainly helpful in thinking through the inclusion and exclusion criteria.

Comment 3:

Clarification is required of how the researchers plan to identify relevant ‘grey’ unpublished literature and I would advise the authors to make clear the exact range of information sources that will be utilised to locate CPGs concerning RM to allow a reproducible literature search.  However, the PICAR statement in Table 1, also identifies that unpublished CPGs will be excluded. This is confusing given the plan to locate unpublished literature sources and this inconsistency should be addressed. 

*Response:*

We have removed reference to “unpublished” after “grey” literature to avoid confusion between the search strategy and the PICAR statement. Grey literature will be used to identify CPGs that may be eligible for inclusion. We will use bibliographic databases (Web of Science, Scopus and Open Grey), as well as guideline repositories (detailed in Table 2) and the websites of professional bodies/organisations (detailed in Table 3), to identify grey literature including conference proceedings and/or technical reports. These, along with the other databases mentioned, comprise the full range of information sources used to locate CPGs. While search terms are presented in Table 4, the full search strategy will be published alongside the paper outlining the findings of this systematic review. 

We have added the following sentence to the “information sources” section to clarify what we mean by grey literature: “Searches of Web of Science, Scopus and Open Grey, as well as guideline repositories and the websites of professional bodies/organisations, will facilitate the identification of grey literature – such as conference proceedings and/or technical reports – which may contain information about potentially eligible CPGs.”

Comment 4:

I was pleased to see the protocol was developed in conjunction with a Pregnancy Loss Patient Representative and encourage the prompt establishment of a PPI group to support this research.

*Response:*

Thank you; this aspect of the work is incredibly important to us. We have since established our PPI group.

Comment 5:

Conclusion: I assume the overall aim of the systematic review is to develop a CPG on RM to promote consistent and standardized care for couples with RM in Ireland, but the actual planned outcome is not clearly articulated and it would be useful to have clarification of this.

*Response:*

As noted in the protocol, this study is a first step in informing efforts to promote the optimisation and standardisation of RM care. This review is being conducted as part of a larger project titled “REcurrent miscarriage: Evaluating CURRENT services (RE:CURRENT)” which is looking at service provision in Ireland. The findings from the systematic review will be used by the RE:CURRENT Team to inform this evaluation; however, we did not detail this within the protocol as the findings of this systematic review will have relevance to the field in general, not just the RE:CURRENT Team. A national guideline is not a planned output of this project ; however, evidence gathered during the project may aid future development of a national guideline.

# Peer review: Reviewer response for version 1

**DOI:** 10.21956/hrbopenres.14119.r27846

**Published:** 2020-09-15

**Authors:** Emma Rasmark Roepke

**Affiliations:** 1 Department of Obstetrics and Gynecology, Institute of Clinical Sciences Lund, Skåne University Hospital, Lund University, Lund, Sweden

The aim of this systematic review protocol is to identify and describe published clinical practice guidelines for management, diagnostics and/or follow-up of women with RM. The authors will use an electronic database search to identify published guidelines in English within the last 20 years in high-income countries.

 This study aims to assess a standardisation of RM care. Overall, the protocol is well described with an appropriate study design.

 There are some concerns:

- The PICAR states that only published guidelines will be included though "grey litteature" will also be serached for. Can the authors explaine this further?
- Why is GRADE system not used instead of AGREE?
- How will "low quality" guidelines be compared with "high quality"? How will different quality be implented in the en result (a new guideline)?
- What will the end results be from this expansive research? Is it a national guideline in Ireland?

Is the study design appropriate for the research question?

Partly

Is the rationale for, and objectives of, the study clearly described?

Yes

Are sufficient details of the methods provided to allow replication by others?

Yes

Are the datasets clearly presented in a useable and accessible format?

Yes

Reviewer Expertise:

Recurrent pregnancy loss

I confirm that I have read this submission and believe that I have an appropriate level of expertise to confirm that it is of an acceptable scientific standard, however I have significant reservations, as outlined above.

# Response

**Published:** 2020-10-02

**Authors:** Marita Hennessy

**Affiliations:** University College Cork, Ireland

General comment:

The aim of this systematic review protocol is to identify and describe published clinical practice guidelines for management, diagnostics and/or follow-up of women with RM. The authors will use an electronic database search to identify published guidelines in English within the last 20 years in high-income countries. This study aims to assess a standardisation of RM care. Overall, the protocol is well described with an appropriate study design. There are some concerns.

*Response:*

Thank you for taking the time to review and provide considered feedback on our protocol paper.

Comment 1:

The PICAR states that only published guidelines will be included though "grey literature" will also be searched for. Can the authors explain this further?

*Response:*

We have removed reference to “unpublished” after “grey” literature to avoid confusion between the search strategy and the PICAR statement. Grey literature will be used to identify CPGs that may be eligible for inclusion. We will use bibliographic databases (Web of Science, Scopus and Open Grey), as well as guideline repositories (detailed in Table 2) and the websites of professional bodies/organisations (detailed in Table 3), to identify grey literature including conference proceedings and/or technical reports. These, along with the other databases mentioned, comprise the full range of information sources used to locate CPGs. While search terms are presented in Table 4, the full search strategy will be published alongside the paper outlining the findings of this systematic review. 

We have added the following sentence to the “information sources” section to clarify what we mean by grey literature: “Searches of Web of Science, Scopus and Open Grey, as well as guideline repositories and the websites of professional bodies/organisations, will facilitate the identification of grey literature – such as conference proceedings and/or technical reports – which may contain information about potentially eligible CPGs.”

Comment 2:

Why is GRADE system not used instead of AGREE?  

*Response:*

We are using AGREEII to assess the quality of included CPGs, across the six AGREEII domains. We are not using the GRADE system as we are not assessing the evidence behind the recommendations within each guideline. We are, however, extracting and noting the system of rating evidence used by each CPG. We note within the protocol that “The levels of evidence associated with the recommendations within each CPG will be reported, and quality assessment rating system used; we will not attempt to standardise evidence ratings across CPGs.”

Comment 3:

How will "low quality" guidelines be compared with "high quality"? How will different quality be implemented in the end result (a new guideline)?

*Response:*

We will report the AGREEII scores, by domain, for each CPG. In the narrative synthesis, we will describe CPGs, and recommendations therein, taking account of this quality appraisal. The aim of the review is to identify and appraise CPGs, and describe recommendations therein. We do not aim to develop a CPG from this work (see response 4 for further information).

Comment 4:

What will the end results be from this expansive research? Is it a national guideline in Ireland?

*Response:*

As noted in the protocol, this study is a first step in informing efforts to promote the optimisation and standardisation of RM care. This review is being conducted as part of a larger project titled “REcurrent miscarriage: Evaluating CURRENT services (RE:CURRENT)” which is looking at service provision in Ireland. The findings from the systematic review will be used by the RE:CURRENT Team to inform this evaluation; however, we did not detail this within the protocol as the findings of this systematic review will have relevance to the field in general, not just the RE:CURRENT Team.  A national guideline is not a planned output of this project; however, evidence gathered during the project may aid future development of a national guideline.

# Peer review: Reviewer response for version 1

**DOI:** 10.21956/hrbopenres.14119.r27847

**Published:** 2020-09-07

**Authors:** Jennifer Kaiser

**Affiliations:** 1 Department of Obstetrics and Gynecology, University of Utah School of Medicine, Salt Lake City, UT, USA

This protocol outlines a systematic review of English language, high-income country CPGs for recurrent pregnancy loss in the first trimester. The intention is to identify and describe published CPGs in order to move towards standardization of management, investigation, and follow-up of RM. The strengths of this protocol include its registration with PROSPERO, utilization of a specialist librarian, and adherence to the PRISMA-P guidelines.

- The "grey" literature is denoted as being "unpublished" in the protocol. However, in the PICAR statement, it states that unpublished material will be excluded. Please clarify this seeming inconsistency. Further detail on the "range of information sources" used to locate CPGs would improve reproducibility.
- A lingering question is what the final product of this protocol will be. The aim is to identify, appraise, and describe CPGs with the intent of promoting standardization, but ultimately will this information be use to create a national CPG for Ireland? Or some other consensus statement? A clearer statement on its final uses would strengthen the rationale for this review further.

Is the study design appropriate for the research question?

Yes

Is the rationale for, and objectives of, the study clearly described?

Yes

Are sufficient details of the methods provided to allow replication by others?

Yes

Are the datasets clearly presented in a useable and accessible format?

Not applicable

Reviewer Expertise:

Early pregnancy loss, miscarriage management

I confirm that I have read this submission and believe that I have an appropriate level of expertise to confirm that it is of an acceptable scientific standard.

# Response

**Published:** 2020-09-23

**Authors:** Marita Hennessy

**Affiliations:** University College Cork, Ireland

Thank you for taking the time to review and provide feedback on our protocol paper.

Comment 1:

The "grey" literature is denoted as being "unpublished" in the protocol. However, in the PICAR statement, it states that unpublished material will be excluded. Please clarify this seeming inconsistency. Further detail on the "range of information sources" used to locate CPGs would improve reproducibility.

*Response:*

We have removed reference to “unpublished” after “grey” literature to avoid confusion between the search strategy and the PICAR statement. Grey literature will be used to identify CPGs that may be eligible for inclusion. We will use bibliographic databases (Web of Science, Scopus and Open Grey), as well as guideline repositories (detailed in Table 2) and the websites of professional bodies/organisations (detailed in Table 3), to identify grey literature including conference proceedings and/or technical reports. These, along with the other databases mentioned, comprise the full range of information sources used to locate CPGs. While search terms are presented in Table 4, the full search strategy will be published alongside the paper outlining the findings of this systematic review. 

We have added the following sentence to the “information sources” section to clarify what we mean by grey literature: “Searches of Web of Science, Scopus and Open Grey, as well as guideline repositories and the websites of professional bodies/organisations, will facilitate the identification of grey literature – such as conference proceedings and/or technical reports – which may contain information about potentially eligible CPGs.”

Comment 2:

A lingering question is what the final product of this protocol will be. The aim is to identify, appraise, and describe CPGs with the intent of promoting standardisation, but ultimately will this information be use to create a national CPG for Ireland? Or some other consensus statement? A clearer statement on its final uses would strengthen the rationale for this review further.

*Response:*

As noted in the protocol, this study is a first step in informing efforts to promote the optimisation and standardisation of RM care. This review is being conducted as part of a larger project titled “REcurrent miscarriage: Evaluating CURRENT services (RE:CURRENT)” which is looking at service provision in Ireland. The findings from the systematic review will be used by the RE:CURRENT Team to inform this evaluation; however, we did not detail this within the protocol as the findings of this systematic review will have relevance to the field in general, not just the RE:CURRENT Team.

# Peer review: Reviewer response for version 1

**DOI:** 10.21956/hrbopenres.14119.r27360

**Published:** 2020-05-18

**Authors:** Merel M. J. van den Berg

**Affiliations:** 1 Centre for Reproductive Medicine, Amsterdam University Medical Centres, University of Amsterdam, Amsterdam, The Netherlands

Summary

 This systematic review identifies and describes published clinical practice guidelines for the management, diagnostics and/or follow-up of women with RM. This studies focuses on high-income countries. Electronic databases, selected guideline repositories and the websites of professional societies will be searched to identify guidelines within the last 20 years.

 This study is the first step for the optimisation and standardisation of RM care.

 Overall, the protocol is clearly described. The aim of the study is clear with an appropriate study design.

 The only thing what is unclear for me is how the researchers will search for the so-called grey literature. They state that 'We will therefore use a range of information sources to locate CPGs concerning RM.' What do they mean by that? How will they do that? This needs more clarification.

Is the study design appropriate for the research question?

Yes

Is the rationale for, and objectives of, the study clearly described?

Yes

Are sufficient details of the methods provided to allow replication by others?

Partly

Are the datasets clearly presented in a useable and accessible format?

Yes

Reviewer Expertise:

Early pregnancy.

I confirm that I have read this submission and believe that I have an appropriate level of expertise to confirm that it is of an acceptable scientific standard.

# Response

**Published:** 2020-09-23

**Authors:** Marita Hennessy

**Affiliations:** University College Cork, Ireland

Thank you for taking the time to review and provide feedback on our protocol paper.

Comment 1:

The only thing what is unclear for me is how the researchers will search for the so-called grey literature. They state that 'We will therefore use a range of information sources to locate CPGs concerning RM.' What do they mean by that? How will they do that? This needs more clarification.

*Response:*

Grey literature will be used to identify CPGs that may be eligible for inclusion. We will use academic databases (Web of Science, Scopus and Open Grey), as well as guideline repositories (detailed in Table 2) and the websites of professional bodies/organisations (detailed in Table 3), to identify grey literature including conference proceedings and/or technical reports. These, along with the other databases mentioned, comprise the full range of information sources used to locate CPGs. While search terms are presented in Table 4, the full search strategy will be published alongside the paper outlining the findings of this systematic review. 

We have added the following sentence to the “information sources” section to clarify what we mean by grey literature: “Searches of Web of Science, Scopus and Open Grey, as well as guideline repositories and the websites of professional bodies/organisations, will facilitate the identification of grey literature – such as conference proceedings and/or technical reports – which may contain information about potentially eligible CPGs.”
